# Analysis of HER2-Low Breast Cancer in Aotearoa New Zealand: A Nationwide Retrospective Cohort Study

**DOI:** 10.3390/cancers16183204

**Published:** 2024-09-20

**Authors:** Annette Lasham, Reenadevi Ramsaroop, Abbey Wrigley, Nicholas Knowlton

**Affiliations:** 1 Department of Molecular Medicine and Pathology, School of Medical Sciences, Faculty of Medical and Health Sciences, University of Auckland, Auckland 1142, New Zealand; 2 Surgical Pathology Unit, Waitematā Hospital, Te Whatu Ora, Auckland 0610, New Zealand; 3 Canopy Cancer Care, Auckland 1023, New Zealand; 4 Department of Obstetrics, Gynaecology and Reproductive Sciences, School of Medicine, Faculty of Medical and Health Sciences, University of Auckland, Auckland 1142, New Zealand; n.knowlton@auckland.ac.nz

**Keywords:** HER2-low breast cancer, antibody-drug conjugates (ADCs), breast cancer treatment, Aotearoa New Zealand, cancer diagnostics, HER2 testing, personalised medicine, clinical oncology, targeted therapy

## Abstract

**Simple Summary:**

This research is the first comprehensive study in New Zealand to categorise and examine the characteristics of breast cancer based on HER2 status. We explored three HER2 categories: HER2-zero, HER2-low, and HER2-positive, in women diagnosed with invasive breast cancer. Utilising Te Rēhita Mate Ūtaetae (Breast Cancer Foundation NZ National Register), our study analysed data spanning 21 years, revealing that most women underwent HER2 testing. Significantly, many cases previously not recognised as having significant HER2 levels were in fact HER2-low, qualifying them for newer, targeted drug therapies. These findings are particularly crucial as they suggest that newer therapies could benefit a larger segment of patients, notably those with advanced breast cancer; approximately 60% of these women might now benefit from these innovative HER2-targeted treatments. The study underscores the urgent need for standardised HER2 testing to personalise and optimise treatment, enhancing outcomes for patients with invasive breast cancer.

**Abstract:**

Objectives: To perform the first national analysis of demographic and clinicopathological features associated with the HER2 positive, HER2-low, and HER2-zero invasive breast cancers in New Zealand. The study will reveal the proportion of women who may benefit from new HER2-targeted antibody drug conjugate (ADC) therapies. Methods: Utilising data from Te Rēhita Mate Ūtaetae (Breast Cancer Foundation NZ National Register), the study analysed data from women diagnosed with invasive breast cancer over a 21-year period. The HER2 status of tumours was classified into three categories—HER2-zero, HER2-low, HER2-positive. Results: From 2009–2021, 94% of women underwent HER2 testing, with 14% diagnosed with HER2-positive breast cancer. For advanced-stage disease, 38% of those formerly classified as HER2-negative were reclassified as HER2-low. Including HER2-positive breast cancers, this indicates that 60% of women with advanced breast cancer may potentially benefit from the new HER2-directed ADCs (approximately 120 women per year). Conclusions: The findings suggest a significant proportion of women with invasive breast cancer in New Zealand could benefit from new HER2-targeted treatments. There is a need to standardise HER2 testing to enhance personalised treatment and improve outcomes.

## 1. Introduction

Aotearoa New Zealand (NZ) has some of the highest rates of breast cancer in the world [1]. Annually, NZ records approximately 3500 new cases of breast cancer, with a lifetime diagnosis of one in nine women [2]. The mortality rate from breast cancer is around 650 each year [2]. Notably, significant ethnic disparities in breast cancer incidence and mortality rates have been documented, particularly affecting wāhine Māori and Pacific women [3,4,5,6]. Furthermore, compared to Australia, NZ experiences a 16% higher mortality rate from breast cancer, a discrepancy partly attributed to limited access to publicly-funded cancer medications in NZ [7,8,9]. This is relevant as new targeted treatment options are readily available for breast cancer patients internationally.

A good example are drugs targeting tumours over-expressing the ERBB2/HER2 protein (henceforth called HER2). These HER2-positive tumours represent 15–24% of breast cancers [6]. In NZ, trastuzumab was publicly funded for women with HER2-positive advanced breast cancer from July 2005 and, subsequently, for early breast cancer, initially for 9 weeks from July 2007 and later extended to 12 months from December 2008 [10]. Treatment is dependent on companion testing for overexpression of the HER2 protein via immunohistochemistry (IHC) and *ERBB2* (HER2) gene amplification via in situ hybridisation (ISH) and has shown to produce benefits for patients considered HER2-positive as defined by IHC 3+ staining or 2+ staining and who are ISH positive.

Recent clinical trials have demonstrated improved outcomes to HER2 directed therapy in patients with lower levels of HER2 protein expression and/or without gene amplification. These tumours were previously been classified as “HER2 negative” but now are amenable to treatment with HER2-antibody drug conjugates (ADCs), such as trastuzumab deruxtecan (T-DXd) [11]. These tumours are now categorised as HER2-low, distinct from those which have no HER2 expression (now called HER2-zero). The efficacy of this approach was demonstrated in DESTINY-Breast04 for patients with unresectable or metastatic HER2-low breast cancer, which showed significant improvement in progression-free survival and overall survival with T-DXd compared to standard chemotherapy [11]. This now provides a targeted treatment option for patients with both the newly-identified HER2-low and HER2-positive tumours.

The aim of this paper is to perform the first national analysis of demographic and clinicopathological features associated with HER2-positive, HER2-low, and HER2-zero invasive breast cancers across New Zealand, to estimate the proportion of women who stand to benefit from new targeted therapies for HER2-low and HER2-positive tumours.

## 2. Materials and Methods

### 2.1. Data Source

Data extracted from Te Rēhita Mate Ūtaetae (Breast Cancer Foundation NZ National Register) included women diagnosed with breast cancer in Aotearoa New Zealand (NZ) between 2000–2021. Te Rēhita is an opt-out registry that contains prospectively collected information on breast cancer diagnosed in the Auckland and Waikato regions from 2000, in the Christchurch region from 2009, the Wellington region from 2010, and nationally from 2020. The opt-out rate is 0.1% since 2012 [6]. Ethnicity information in Te Rēhita is populated from Ministry of Health data, and this study followed the assignment of persons to single ethnicity groups as described in [6].

### 2.2. Variables

**Figure 1:**
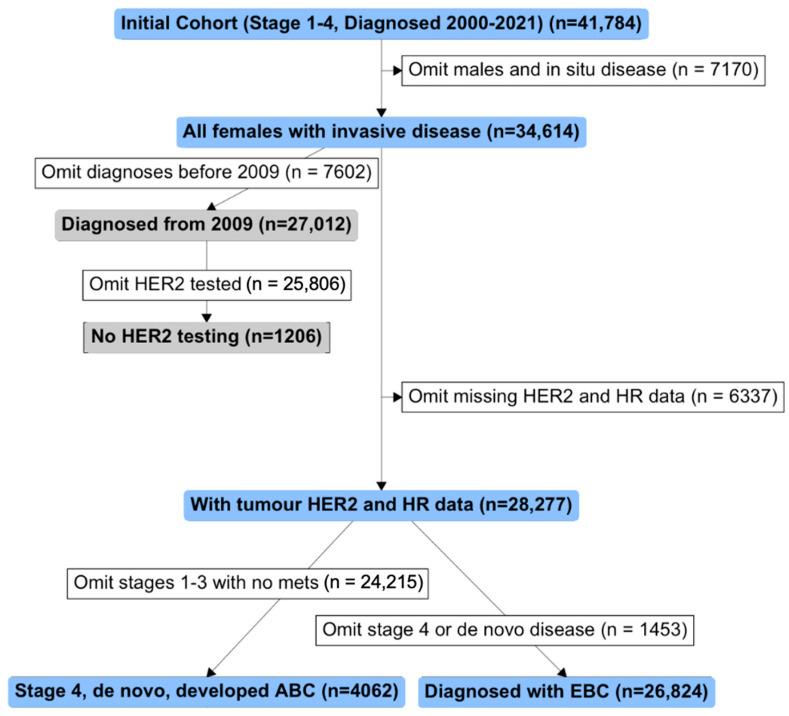
Flowchart showing inclusion (blue/grey boxes) and exclusion (white boxes) criteria for this study. Men diagnosed with breast cancer (*n* = 287) and women diagnosed with in situ disease (stage 0, n = 6528) were omitted at the outset. Only for the purpose of investigating which women did and did not receive HER2 tumour testing over time were distinct cohorts defined comprising women with invasive breast cancer with or without HER2 tumour data (grey boxes). For the main analyses (shown with blue boxes), all women with invasive disease, with tumour hormone receptor (HR) and HER2 data (either HER2 positive by FISH, or with HER2 immunohistochemistry staining data) were included and then further analysed in cohorts of advanced breast cancer (ABC; diagnosed with Stage 4 disease, de novo disease or developed advanced breast cancer) and early breast cancer (EBC; diagnosed with Stages 1–3 disease). Note that 2625 women diagnosed with EBC developed advanced breast cancer, so these women are included in both ABC and EBC cohorts.

Only women with invasive breast cancer (AJCC7 stages 1–4) were eligible for this analysis (Figure 1). Women with invasive tumours that had missing hormone receptor data, no HER2 data, or that were HER2 FISH negative with no HER2 immunohistochemistry (IHC) data were omitted from subsequent analyses of HER2 status (19%). Hormone receptor status of tumours was defined as follows: hormone receptor positive (HR+) was oestrogen receptor (ER) and/or progesterone receptor (PR) positive, and hormone receptor negative (HR−) was ER and PR negative (following 1% cut-off guidelines for ER/PR). HER2 status was defined as HER2-positive if the tumour was either FISH positive or had an IHC staining of 3+. HER2 negative tumours were defined as either FISH negative and/or having an IHC staining of 0, 1+, or 2+. HER2-low tumours were defined as FISH negative and/or having an IHC staining of 1+ or 2+. HER2-zero (HER2-0) tumours were defined as having an IHC staining of 0. Over 76% of cases included in this analysis underwent a secondary review by an independent pathologist during multidisciplinary meetings, ensuring that the assessments of hormone receptor and HER2 status were rigorously peer-reviewed. Although these reviews were conducted within the same pathology laboratory, they adhered to strict quality control measures to maintain accuracy and reliability of the diagnostic assessments.

### 2.3. Statistical Analyses

Data were summarised in counts (N) and percentages for categorical data. Differences of proportions between two or more subgroups were tested with chi-squared tests. A nominal alpha of 0.01 is considered statistically significant. All analyses were performed in R 4.3.1. Comparative analyses between advanced and early breast cancer cohorts were omitted owing to the lack of mutual exclusivity between these groups.

### 2.4. Ethics

Te Rēhita Mate Ūtaetae operates under the NZ Health and Disability Ethics Committee approval (16/NTA/139/AM03), privacy, and health legislation and Treaty of Waitangi principles [6]. This specific study was approved by the Auckland Health Research Ethics Committee (AH2800).

## 3. Results

### 3.1. How Many Women Had HER2 Testing and Who Gets Tested?

The HER2 test is a companion diagnostic test and is required to decide on treatment regimes. The proportion of women who underwent HER2 tumour testing via either immunohistochemistry or ISH starting from the year 2009 was analysed. This showed that approximately 94–96% of women had their breast tumours tested for HER2 (Table 1).

Of those tested, approximately 13–15% of women were diagnosed with HER2 positive breast cancer. Most regions performed 60–70% of HER2 testing on the excised lesion, except Midland, where nearly 90% of HER2 testing was performed on the core biopsy sample (Table 2).

Analysis by age at diagnosis showed that 2.3% of women under 70 years did not receive HER2 testing, but this was considerably higher (at 13.1%) for women diagnosed at 70 years and over (Table 3). A total of 23.1% of women under 45 years old had HER2 positive breast tumours, in contrast to 13.3% of women diagnosed between 45–69 years.

Approximately 6% of European women did not have HER2 testing, almost double that of wāhine Māori (Table 4). As previously reported [6,12], a significantly higher proportion of Pacific women had HER2 positive breast cancers (21.5%) than European women (12%).

Analysis by region of domicile at diagnosis revealed that 3–4% of women were not tested for HER2 status across all regions, with the exception of the Southern region, where over 10% of women’s tumours were untested for HER2 status (Table 5). The proportion of women with HER2 positive breast tumours was higher in the Northern and Southern regions.

### 3.2. Who Was Not Tested?

Subsequent analysis was performed to understand which women did not have their tumours tested for HER2 status. Sixty-seven per cent of these women were over 69 years old when diagnosed with breast cancer, and 81% were European women (Table 6). Regionally, the highest numbers of tumours that were not tested were in the Northern and Southern regions. Thirty-nine per cent had stage 1 disease, while 27% had missing stage information but were documented as having invasive disease from a core biopsy sample (Table 6).

### 3.3. What Is the Proportion of HER2-Positive, HER2-Low, and HER2-Zero Breast Cancers in New Zealand?

Te Rēhita Mate Ūtaetae collects detailed data around the reporting of HER2 results and this enabled the HER2 negative tumours to be stratified into HER2-zero and HER2-low (acknowledging limitations [13,14,15]). A further stratification via HR status divided the patient groups into HR positive and HR negative, which were then analysed based on whether the patients had advanced or early-stage breast cancer, allowing for a more detailed understanding of treatment needs across different stages of the disease.

#### 3.3.1. Advanced Breast Cancer

**Figure 2:**
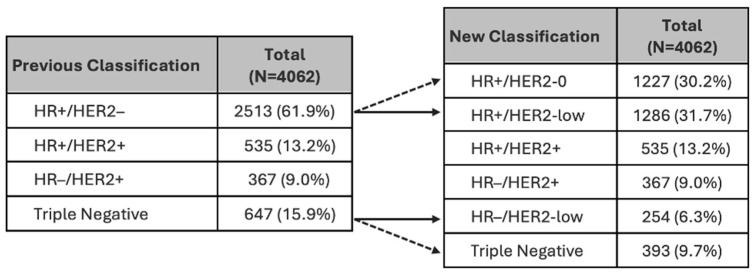
Analysis of women with advanced breast cancer by tumour receptor status, prior to and after re-categorisation of HER2 negative tumours. HR = hormone receptor. Note: Arrows illustrate the re-categorised patients according to the updated classification system. Triple Negative = HR−/HER2-0 in the right table.

Thirty eight percent of women with advanced breast cancer could be re-categorised as HER2-low, 51% of these were HR positive, and 39% HR negative (Figure 2).

Analysis of the six HER2 subclasses by demographic and pathological features for advanced disease revealed notable differences. Women diagnosed before the age of 45 had the highest proportions of HER2 positive breast cancers, making this younger age group the most likely to benefit from HER-targeting ADC treatments (Table 7). No statistical relationship was found between HER2-low status and age group.

By ethnicity, Pacific women displayed the highest proportion of HER2+ breast cancers (Table 8). The incidence of HER2-low cancers was similar across all ethnic groups. While wāhine Māori and Pacific women would benefit disproportionately from access to HER2-targeting therapies, this advantage is primarily attributed to the higher prevalence of HER2-positive cases rather than an increased occurrence of HER2-low tumours.

Analysis by tumour pathology revealed that HR−/HER2-low tumours were predominantly grade 3, while HR+/HER2-low were primarily grades 1 or 2 (Table 9). Interestingly, the distribution of tumour sizes showed no significant variation across the subclasses.

#### 3.3.2. Early Breast Cancer

**Figure 3:**
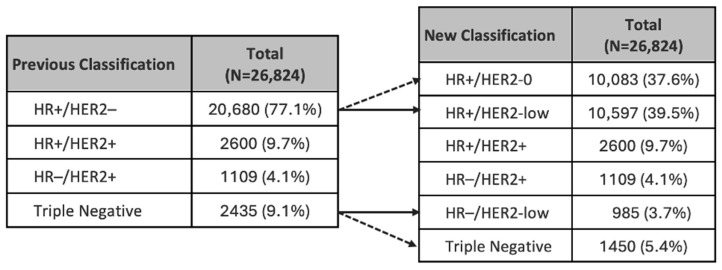
Analysis of women with early breast cancer by tumour receptor status, prior to and after reclassification of HER2 negative tumours. HR = hormone receptor. Note: Arrows illustrate the reclassification of patients according to the updated classification system. Triple Negative = HR−/HER2-0 in the right table.

Although clinical trials have not yet been conducted for early-stage breast cancer, we examined what proportion of women had HER2-low tumours. Our analysis showed that 43% of women with early breast cancer could be reclassified as having HER2-low disease. Specifically, this includes 51% of those previously classified as HR+/HER2-negative and 40% of those previously identified as triple-negative early breast cancers (Figure 3).

Analysis of the various receptor subclasses by women’s demographics showed that those diagnosed before 45 years of age had the highest proportions of tumours with poor prognostic features (HR+/HER2+ and HR− tumours at 16% and 20%, respectively), compared to women diagnosed at or above 45 years of age (who had 9% of HR+/HER2+ and 12% HR− tumours) (Table 10).

By ethnicity, 22% of Pacific women were diagnosed with HER2-positive breast cancers, compared to 13–16% among women of other ethnicities (Table 11). Apart from this, the distribution of each subclass was similar across ethnic groups. Irrespective of the age at diagnosis or ethnicity, 40% of women with tumours previously classified as triple negative would now be classified as HR−/HER2-low.

Analysis of the subclasses by clinicopathological features revealed that HR+/HER2-low and HR+/HER2-zero breast cancers had similar characteristics: 59–60% were stage 1, 63% had N0 nodal status, 50–51% were grade 2 tumours, and 55% of tumours measured 20mm or smaller (Table 12). Similarly, HR−/HER2-low and triple-negative breast cancers also showed comparable features with 48% being stage 1, 43–44% stage 2, 77–78% grade 3 tumours, and 48–49% measuring 21–50 mm in size, although nodal status was more variable (Table 12).

## 4. Discussion

The introduction of trastuzumab deruxtecan (T-DXd) marks a significant advancement in the personalised treatment landscape for women with breast cancer, particularly for those classified as having HER2-low tumours [11]. This study provides the first analysis of HER2 testing patterns of women diagnosed with breast cancer in Aotearoa New Zealand, and the proportion of women who might benefit from these new drugs.

This analysis showed that, from 2009, over 94% of women diagnosed with invasive breast cancer had HER2 testing, following international standards and establishing the critical role of HER2 testing in guiding therapies offered to NZ women. However, considerably lower testing rates were observed for women diagnosed after 70 years of age, of these 85% were European. These findings are consistent with a previous NZ study showing that chemotherapy was given to only 5% of women diagnosed with invasive breast cancer from age 70 [16].

The data also showed that women diagnosed with advanced breast cancers before the age of 45 exhibited the highest proportions of HER2+ tumours. Additionally, wāhine Māori and Pacific women with advanced disease displayed higher levels of HR+/HER2+ tumours. Notably, Pacific women recorded the highest proportion of HER2+ tumours across all disease stages, confirming previous findings [6,12]. For these groups, especially those with advanced breast cancer, access to new HER2-targeting ADCs could significantly enhance treatment efficacy and patient outcomes. Interestingly, our analysis revealed no significant differences in the age or ethnicity of patients with HER2-low tumours, suggesting that HER2-low status may manifest independently of these demographic factors.

The new stratification of breast cancers from HER2-negative to HER2-low or HER2-zero subclasses (noting that HR−/HER2-zero = triple negative) in our cohort has shown that HER2-low subclasses comprise over 50% of HR+/HER2-negative and ~40% of previousy classified triple negative breast cancers for both advanced and early breast cancers. Using this information, these new therapies could provide treatment options for 32% of advanced breast cancers previously classified as HER2-negative. In 2022, the FDA approved the use of T-DXd for unresectable or metastatic HER2-low breast cancer as second-line therapy after chemotherapy or if there was disease recurrence during or within six months of completing adjuvant chemotherapy [17]. In addition to HER2-positive advanced breast cancer, this would be approximately 120 women per year, based on 2020 and 2021 data, that would be eligible in NZ.

At the time of writing, there are a number of clinical trials underway, evaluating T-DXd as a first line treatment, alone or in combination with pertuzumab, for HER2-positive advanced breast cancer (DESTINY-Breast07) [18]. DESTINY-Breast06 directly compares standard-of-care chemotherapy to T-DXd for HR+/HER2-low or HR+/HER2-ultralow (defined as IHC 0 with minimal (<1+) membrane staining) advanced breast cancers after one or more lines of endocrine therapy. To date, both of these trials have shown improved outcomes for patients given T-DXd [19].

For future applications of HER2-ADCs for early-stage HER2-low breast cancer, in our opinion, the most likely candidates are those with triple negative disease who are now defined as HR−/HER2-low. These women would then have a targeted therapy available, potentially transforming their treatment options. These drugs could serve as a targeted therapy for 40% of women formerly diagnosed with early triple negative breast cancer, now re-categorised as HER2-low, representing approximately 48 women per year in NZ based on 2020 and 2021 data.

Limitations: the main limitation is the classification of HER2-low and HER2-zero groups from the recorded HER2 immunohistochemistry data. As many studies by pathologists have emphasised, there is evidence of under-identification and over-identification of HER-2 staining level, in part because this level of detail has not previously been required; this test was designed to produce a binary output—HER2-positive or HER2-negative [13,15]. In addition, with potential heterogeneity across a tumour sample and variable staining and reporting between labs, the stratification of our cohort into HER2-low and HER2-zero groups may not be robust [13,15]. A further limitation is the retrospective design of our study, which may affect the precision of HER2-low case identification. While a secondary review was implemented for over 76% of cases, future prospective studies with standardised HER2 testing protocols would be needed to further validate these findings. These limitations apply going forward, hence the international efforts to generate standardised testing protocols to ensure reliable and actionable HER2 status determination [14,20]. Future advancements in diagnostic technologies aim to reduce these inconsistencies, providing a more reliable basis for treatment decisions.

## 5. Conclusions

The potential impact of these therapies for breast cancer in NZ is considerable, especially when considering the current limitations in drug funding and approval timelines by Pharmac [7,9]. An Australian study showed how access to systemic therapies saved the lives of women diagnosed with breast cancer [21]; therefore, ensuring access to these new therapies could help mitigate the observed excess mortality in breast cancer patients in NZ compared to Australia. To prepare for the future availability of HER2-low targeting therapies, NZ pathologists are undergoing training to accurately classify HER2-low tumours. This proactive training strategy enhances diagnostic precision and ensures that NZ is prepared to effectively implement these advanced treatment options when they become available. Prospective studies are warranted to further investigate HER2-low breast cancer identification, particularly as pathologists in NZ continue to receive specialised training. These future studies could provide more standardised and reproducible assessments, especially in light of emerging HER2-targeted treatments.

In conclusion, the potential to treat a wider spectrum of breast cancers with targeted therapies like T-DXd could transform the therapeutic landscape in NZ. Ensuring that these benefits reach all segments of the population will require concerted efforts in medical research, healthcare policy, and clinical practice. Our study sets the groundwork for these developments, advocating for a paradigm shift in how breast cancer is treated in the national context.

## Figures and Tables

**Table 1:** Numbers and proportions of women who had tumour HER2 testing and testing results by their year of diagnosis.

	2009–2011 (*n* = 4443)	2012–2014 (*n* = 5538)	2015–2017 (*n* = 5917)	2018–2020 (*n* = 7646)	2021 (*n* = 3468)
HER2 negative *n* (%)	3586 (80.7%)	4473 (80.8%)	4771 (80.6%)	6382 (83.5%)	2865 (82.6%)
HER2 positive *n* (%)	606 (13.6%)	743 (13.4%)	873 (14.8%)	950 (12.4%)	431 (12.4%)
Not tested *n* (%)	251 (5.6%)	322 (5.8%)	273 (4.6%)	314 (4.1%)	172 (5.0%)

**Table 2:** Sample used for HER2 testing by geographical region of diagnosis (since 2009).

	Northern (*n* = 12,141)	Midland (*n* = 4325)	Central (*n* = 4408)	Southern (*n* = 4889)	*p* Value
Core biopsy *n* (%)	4924 (40.6%)	3841 (88.8%)	1333 (30.2%)	1913 (39.1%)	<0.001
Excision *n* (%)	7217 (59.4%)	484 (11.2%)	3075 (69.8%)	2976 (60.9%)	

**Table 3:** Numbers and proportions of women (diagnosed from 2009) who had tumour HER2 testing by their age at diagnosis.

	≤44 Years (*n* = 3122)	45–69 Years (*n* = 17,383)	≥70 Years (*n* = 6507)	*p* Value
HER2 negative *n* (%)	2332 (74.7%)	14,666 (84.4%)	5079 (78.1%)	
HER2 positive *n* (%)	722 (23.1%)	2307 (13.3%)	574 (8.8%)	<0.001
Not tested *n* (%)	68 (2.2%)	410 (2.4%)	854 (13.1%)	

**Table 4:** Numbers and proportions of women (diagnosed from 2009) who had tumour HER2 testing by their ethnicity.

	Māori (*n* = 3131)	Pacific (*n* = 1765)	Asian (*n* = 2260)	Other (*n* = 404)	European (*n* = 19,452)	*p* Value
HER2 negative *n* (%)	2560 (81.8%)	1313 (74.4%)	1828 (80.9%)	329 (81.4%)	16,047 (82.5%)	
HER2 positive *n* (%)	476 (15.2%)	379 (21.5%)	350 (15.5%)	61 (15.1%)	2337 (12.0%)	<0.001
Not tested *n* (%)	95 (3.0%)	73 (4.1%)	82 (3.6%)	14 (3.5%)	1068 (5.5%)	

**Table 5:** Numbers and proportions of women (diagnosed from 2009) who had tumour HER2 testing by the region in which they were diagnosed.

	Northern (*n* = 12,592)	Midland (*n* = 4405)	Central (*n* = 4549)	Southern (*n* = 5466)	*p* Value
HER2 negative *n* (%)	10,408 (82.7%)	3668 (83.3%)	3868 (85.0%)	4133 (75.6%)	
HER2 positive *n* (%)	1735 (13.8%)	583 (13.2%)	531 (11.7%)	754 (13.8%)	0.007
Not tested *n* (%)	449 (3.6%)	154 (3.5%)	150 (3.3%)	579 (10.6%)	

**Table 6:** Description of those women not receiving testing for tumour HER2 status, from 2009.

	Not Tested (*n* = 1206)
Age at diagnosis *n* (%)	
≤44 years	57 (4.7%)
45–69 years	336 (27.9%)
≥70 years	813 (67.4%)
Ethnicity *n* (%)	
Māori	74 (6.1%)
Pacific	69 (5.7%)
Asian	71 (5.9%)
Other	14 (1.2%)
European	978 (81.1%)
Region at Diagnosis *n* (%)	
Northern	431 (35.7%)
Midland	76 (6.3%)
Central	136 (11.3%)
Southern	563 (46.7%)
TNM Stage ^1^ *n* (%)	
1	465 (38.6%)
2	245 (20.3%)
3	55 (4.6%)
4	120 (10.0%)
None recorded	321 (26.6%)

^1^ AJCC7 staging.

**Table 7:** Description of women with advanced breast cancer by HER2 subclasses by age at diagnosis.

	≤44 Years (*n* = 884)	45–69 Years (*n* = 2126)	≥70 Years (*n* = 1052)	*p* Value
Receptor status *n* (%)				
HR+/HER2-0	231 (26.1%)	634 (29.8%)	362 (34.4%)	ns ^1^
HR+/HER2-low	266 (30.1%)	671 (31.6%)	349 (33.2%)	
HR+/HER2+	148 (16.7%)	275 (12.9%)	112 (10.6%)	
HR−/HER2+	104 (11.8%)	201 (9.5%)	62 (5.9%)	
HR−/HER2-low	49 (5.5%)	138 (6.5%)	67 (6.4%)	
Triple Negative	86 (9.7%)	207 (9.7%)	100 (9.5%)	
**Total that would benefit from HER-targeting ADC Treatments** *n* (%)	567 (64%)	1285 (60%)	590 (56%)	

^1^ ns = not significant.

**Table 8:** Description of women with advanced breast cancer by HER2 subclass by ethnicity.

	Māori (*n* = 473)	Pacific (*n* = 420)	Asian (*n* = 286)	European (*n* = 2759)	*p* Value
Receptor status *n* (%)					
HR+/HER2-0	133 (28.1%)	119 (28.3%)	80 (28.0%)	866 (31.4%)	0.010
HR+/HER2-low	155 (32.8%)	138 (32.9%)	87 (30.4%)	866 (31.4%)	
HR+/HER2+	74 (15.6%)	80 (19.0%)	39 (13.6%)	327 (11.9%)	
HR−/HER2+	47 (9.9%)	54 (12.9%)	34 (11.9%)	212 (7.7%)	
HR−/HER2-low	28 (5.9%)	13 (3.1%)	15 (5.2%)	190 (6.9%)	
Triple Negative	36 (7.6%)	16 (3.8%)	31 (10.8%)	298 (10.8%)	
**Total that would benefit from HER-targeting ADC Treatments** *n* (%)	304 (64%)	285 (67%)	176 (62%)	1595 (58%)	

124 women with “other ethnicity” omitted from this table due to low numbers across the subclasses.

**Table 9:** Description of advanced tumours by HER2 subclasses—histopathological grade and tumour size.

	HR+/ HER2-0 (*n* = 1227)	HR+/ HER2-Low (*n* = 1286)	HR+/ HER2+ (*n* = 535)	HR−/ HER2+ (*n* = 367)	HR−/ HER2-Low (*n* = 254)	Triple Negative (*n* = 393)	*p* Value
Tumour grade *n* (%)							
1 or 2 ^1^	592 (48.2%)	615 (47.8%)	129 (24.1%)	47 (12.8%)	34 (13.4%)	58 (14.7%)	
3	335 (27.3%)	401 (31.2%)	250 (46.7%)	217 (59.1%)	187 (73.6%)	287 (73.0%)	<0.001
None recorded	300 (24.5%)	270 (21.0%)	156 (29.1%)	103 (28.1%)	33 (13.0%)	48 (12.2%)	
Tumour size *n* (%)							
≤20 mm	192 (15.6%)	216 (16.8%)	92 (17.2%)	74 (20.2%)	49 (19.3%)	87 (22.1%)	
21–50 mm	565 (46.0%)	571 (44.4%)	219 (40.9%)	149 (40.6%)	131 (51.6%)	204 (51.9%)	ns ^2^
>50 mm	172 (14.0%)	214 (16.6%)	73 (13.6%)	51 (13.9%)	41 (16.1%)	59 (15.0%)	
None recorded	298 (24.3%)	285 (22.2%)	151 (28.2%)	93 (25.3%)	33 (13.0%)	43 (10.9%)	

^1^ Tumour grades 1 and 2 were combined because of low numbers for some subclasses. ^2^ ns = not significant.

**Table 10:** Description of women diagnosed with early breast cancer by HER2 subclasses by age at diagnosis.

	≤44 Years (*n* = 3512)	45–69 Years (*n* = 17,889)	≥70 Years (*n* = 5423)	*p* Value
Receptor status *n* (%)				
HR+/HER2-0	1050 (29.9%)	6792 (38.0%)	2241 (41.3%)	<0.001
HR+/HER2-low	1219 (34.7%)	7225 (40.4%)	2153 (39.7%)	
HR+/HER2+	553 (15.7%)	1664 (9.3%)	383 (7.1%)	
HR−/HER2+	218 (6.2%)	747 (4.2%)	144 (2.7%)	
HR−/HER2-low	190 (5.4%)	595 (3.3%)	200 (3.7%)	
Triple Negative	282 (8.0%)	866 (4.8%)	302 (5.6%)	

**Table 11:** Description of women diagnosed with early breast cancer by HER2 subclasses by ethnicity.

	Māori (*n* = 2882)	Pacific (*n* = 1743)	Asian (*n* = 2321)	Other (*n* = 435)	European (*n* = 19,443)	*p* Value
Receptor status *n* (%)						
HR+/HER2-0	1143 (39.7%)	669 (38.4%)	829 (35.7%)	148 (34.0%)	7294 (37.5%)	<0.001
HR+/HER2-low	1111 (38.5%)	586 (33.6%)	913 (39.3%)	166 (38.2%)	7821 (40.2%)	
HR+/HER2+	297 (10.3%)	239 (13.7%)	262 (11.3%)	47 (10.8%)	1755 (9.0%)	
HR−/HER2+	131 (4.5%)	148 (8.5%)	107 (4.6%)	22 (5.1%)	701 (3.6%)	
HR−/HER2-low	83 (2.9%)	38 (2.2%)	87 (3.7%)	16 (3.7%)	761 (3.9%)	
Triple Negative	117 (4.1%)	63 (3.6%)	123 (5.3%)	36 (8.3%)	1111 (5.7%)	

**Table 12:** Description of clinicopathological features of early breast cancers by HER2 subclasses.

	HR+/ HER2-0 (*n* = 10,083)	HR+/ HER2-Low (*n* = 10,597)	HR+/ HER2+ (*n* = 2600)	HR−/ HER2+ (*n* = 1109)	HR−/ HER2-Low (*n* = 985)	Triple Negative (*n* = 1450)	*p* Value
TNM stage ^1^ *n* (%)							
1	6002 (59.5%)	6286 (59.3%)	1341 (51.6%)	513 (46.3%)	472 (47.9%)	700 (48.3%)	<0.001
2	3037 (30.1%)	3360 (31.7%)	985 (37.9%)	464 (41.8%)	432 (43.9%)	623 (43.0%)	
3	558 (5.5%)	584 (5.5%)	197 (7.6%)	92 (8.3%)	63 (6.4%)	90 (6.2%)	
None recorded	486 (4.8%)	367 (3.5%)	77 (3.0%)	40 (3.6%)	18 (1.8%)	37 (2.6%)	
Nodal status *n* (%)							
0	6354 (63.0%)	6694 (63.2%)	1474 (56.7%)	565 (50.9%)	663 (67.3%)	937 (64.6%)	<0.001
1	2133 (21.2%)	2442 (23.0%)	665 (25.6%)	282 (25.4%)	177 (18.0%)	305 (21.0%)	
2	561 (5.6%)	586 (5.5%)	218 (8.4%)	121 (10.9%)	72 (7.3%)	86 (5.9%)	
3	310 (3.1%)	318 (3.0%)	133 (5.1%)	85 (7.7%)	40 (4.1%)	52 (3.6%)	
None recorded	725 (7.2%)	557 (5.3%)	110 (4.2%)	56 (5.0%)	33 (3.4%)	71 (4.8%)	
Tumour grade *n* (%)							
1	2972 (29.5%)	2601 (24.5%)	120 (4.6%)	<10 (0.7%)	16 (1.6%)	25 (1.7%)	
2	5041 (50.0%)	5385 (50.8%)	1061 (40.8%)	224 (20.2%)	173 (17.6%)	243 (16.8%)	<0.001
3	1559 (15.5%)	2230 (21.0%)	1314 (50.5%)	807 (72.8%)	764 (77.6%)	1121 (77.3%)	
None recorded	511 (5.0%)	381 (3.6%)	105 (4.1%)	70 (6.3%)	32 (3.2%)	61 (4.2%)	
Tumour size *n* (%)							
≤20 mm	5554 (55.1%)	5779 (54.5%)	1257 (48.3%)	496 (44.7%)	426 (43.2%)	482 (48.9%)	<0.001
21–50 mm	3525 (35.0%)	3915 (36.9%)	1109 (42.7%)	505 (45.5%)	482 (48.9%)	693 (47.8%)	
>50 mm	535 (5.3%)	551 (5.2%)	163 (6.3%)	77 (6.9%)	58 (5.9%)	84 (5.8%)	
None recorded	469 (4.7%)	352 (3.3%)	71 (2.7%)	31 (2.8%)	19 (1.9%)	34 (2.3%)	

^1^ AJCC7 staging.

## Data Availability

The dataset presented in this article is not readily available because this requires approval by the custodians of the data. Requests to access the dataset should be directed to Te Rēhita Mate Ūtaetae.
